# Life-Threatening Complications and Intensive Care of Diffuse Neonatal Hemangiomatosis of the Liver in a One-Month-Old Girl: A Case Report

**DOI:** 10.3390/children13060747

**Published:** 2026-05-27

**Authors:** Regina R. Tukhvatullina, Nune V. Matinyan, Vasiliy A. Akimov, Raisa V. Milutis, Tatiana S. Belysheva, Lana R. Turkia, Ekaterina V. Chufistova, Guzel M. Muftakhova, Julietta A. Karmanova, Anatoly P. Kazantsev, Vera V. Reshetnikova, Timur T. Valiev, Kirill I. Kirgizov, Tatiana V. Gorbunova, Svetlana R. Varfolomeeva, Irina Zh. Shubina, Mikhail V. Kiselevskiy

**Affiliations:** 1 Research Institute of Pediatric Oncology and Hematology, FSBI “N.N. Blokhin National Medical Research Center of Oncology” of the Ministry of Health of Russia, Kashirskoye Sh. 23, Moscow 115522, Russia; r.tukhvatullina@ronc.ru (R.R.T.); n.matinyan@ronc.ru (N.V.M.); v.akimov@ronc.ru (V.A.A.); r.milutis@ronc.ru (R.V.M.); t.belysheva@ronc.ru (T.S.B.); l.turkija@ronc.ru (L.R.T.); e.chufistova@ronc.ru (E.V.C.); g.muftakhova@ronc.ru (G.M.M.); d.karmanova@ronc.ru (J.A.K.); a.kazantsev@ronc.ru (A.P.K.); t.valiev@ronc.ru (T.T.V.); k.kirgizov@ronc.ru (K.I.K.); t.gorbunova@ronc.ru (T.V.G.); s.varfolomeeva@ronc.ru (S.R.V.); 2 Research Institute of Experimental Oncology and Carcinogenesis, FSBI “N.N. Blokhin National Medical Research Center of Oncology” of the Ministry of Health of Russia, Kashirskoye Sh. 24, Moscow 115522, Russia; v.reshetnikova@ronc.ru (V.V.R.); kisele@inbox.ru (M.V.K.)

**Keywords:** infantile hemangioma, hemangiomatosis, infants, β-adrenoblockers, propranolol, life-threatening conditions

## Abstract

**Highlights:**

**What are the main findings?**

- Hemangiomas of internal organs in newborns are a rare and potentially life-threatening condition.
- Patients presenting with life-threatening conditions may require therapy in the intensive care unit and multidisciplinary care involving an oncologist, dermatologist, endocrinologist, or cardiologist.

**What are the implications of the main findings?**

- Currently, oral propranolol suspension is the therapy of choice for children with infantile hemangiomatosis.
- Timely detection and targeted treatment of infantile hemangiomatosis significantly improve the prognosis and quality of life of children.

**Abstract:**

**Background**: An infantile hemangioma (IH) is a benign lesion that develops as a result of pathologically dysregulated proliferation of the endothelial cells of the capillaries, typically appearing within the first three years of life. The disease is found mostly on the skin and—less often—in the internal organs. Although IH generally has a good prognosis and may sometimes undergo spontaneous regression, certain types of IH with a specific form, localization, and size may lead to life-threatening conditions including impairment of vital functions. This determines the need for precise diagnostics and treatment. **Case presentation**: The paper presents a clinical observation of an infant in the first months of life with IH presenting as diffuse hepatic hemangiomatosis and a hemangioma of the left eyebrow area. The IH was associated with life-threatening conditions. However, effective treatment with the non-selective β-adrenoblocker propranolol led to a positive outcome and the resolution of complications. **Conclusions**: This case demonstrates the critical importance of timely diagnosis and treatment for lesions that, while benign, can lead to fatal complications if not addressed promptly.

## 1. Introduction

Infantile hemangiomas (IH) are benign vascular neoplasms that are the most common head and neck tumors in infancy [1]. Although they can occur almost anywhere in the body, the majority are located in the head and neck region. They occur most frequently in premature infants with a body weight of less than 1800 g [2], more often in Caucasian girls.

These benign vascular tumors undergo a phase of rapid proliferation, which may continue up to 8–12 months, then a slow spontaneous involution may begin, lasting 4–7 years; therefore most patients do not need any treatment [1,3]. However, the diffuse hemangiomatosis of internal organs can probably lead to developing severe life-threatening conditions (LTC). Multiple skin hemangiomas in diffuse neonatal hemangiomatosis can combine with lesions in the internal organs, primarily the liver [4]. Diffuse hemangiomas can lead to abdominal compartment syndrome (ACS) due to the associated hepatomegaly, as well as to heart failure (resulting from increased venous return and preload on the right heart) and respiratory failure [5]. Severe respiratory failure and respiratory distress syndrome may occur in case of IH localization in the upper respiratory tract due to the compression of the organs [6,7].

Severe acquired thyroid dysfunction can also occur as a result of type 3 deiodinase overexpression (an enzyme that inactivates thyroid hormones), which leads to increased conversion of thyroxine and triiodothyronine to biologically inactive metabolites (reverse T3 and diiodothyronine T2). Hypothyroidism is consistently observed in diffuse hemangiomas, whereas it is rarely noted in multifocal variants [2,8]. Timely diagnosis of IH and initiation of appropriate therapy are critical to saving a child’s life. First-line therapy for diffuse lesions includes the non-selective β-adrenoblocker propranolol, which has proven effective in the treatment of IH [7,9].

We report a clinical case of examination and treatment of a patient with a complicated IH, which required differential diagnosis from malignant tumors and immediate initiation of the specific treatment and concomitant therapy to manage the LTC. The consent of legal representatives for the publication was obtained.

## 2. Case Presentation

In June 2024 a girl, M., aged 1 month with a bulky formation in the abdominal cavity was admitted to the L.A. Durnov Research Institute of Pediatric Oncology and Hematology of the “N.N.Blokhin National Research Medical Center of oncology” under the Ministry of Health of Russia (hereinafter referred to as the RI PO&H). The girl was born via natural childbirth after an uncomplicated pregnancy. Overall, the pregnancy was uncomplicated, and ultrasound examinations revealed no structural or vascular abnormalities.

The child’s parents noted an increase in the size of the belly from the 15th day of life. An abdominal ultrasound (US) performed at a local clinic demonstrated diffuse hepatic involvement characterized by multiple hypoechoic, round-shaped lesions measuring up to 1.8 cm in diameter. These findings were subsequently confirmed by computed tomography (CT).

After admission to the RI PO&H, the child was hospitalized in the intensive care unit (ICU) taking into account the severe condition probably caused by ACS (the belly was significantly enlarged as a result of severe hepatomegaly; abdominal circumference at admission was 47.5 cm, and the liver protruded 5 cm from the edge of the costal arch). Physical examination revealed hemangiomas of the facial skin at the brow arch on the left, acrocyanosis, tachypnea (respiratory rate over 60/min), a pronounced venous network on the anterior abdominal wall, and a significantly enlarged abdomen, which was dense and tense on palpation.

Laboratory tests showed a high level of α-fetoprotein (AFP) (10,564.1 IU/mL), increased levels of neuron-specific enolase (NSE) (49.93 ng/mL) and β-human chorionic gonadotropin (β-hCG) (0.1 mIU/mL), and thrombocytopenia (48 × 10^9^/L). According to the coagulogram, hypofibrinogenemia was detected (up to 0.4 g/L) along with hypocoagulation in all chains. US examination of the abdominal organs and retroperitoneal space revealed hepatomegaly (oblique vertical dimension of 143 mm) and diffusely heterogeneous parenchyma with multiple areas of reduced echogenicity without clear contours, measuring 10–24 mm in diameter. The ultrasound picture corresponded to hemangiomatosis of the liver.

Evaluation of the hormonal profile revealed an elevated level of thyroid-stimulating hormone (TSH)—90.99 mIU/L (reference range: 0.72–11 mIU/L); a decreased level of free thyroxine (fT4)—44.1 nmol/L (reference range: 69.9–219 nmol/L); and a normal level of free triiodothyronine (fT3)—1.2 nmol/L (reference range: 0.8–2.75 nmol/L). US examination of the thyroid gland did not reveal any abnormalities. The decrease in thyroid function was most likely related to the main disease. Replacement hormone therapy with levothyroxine at a dose of 2 µg/kg/day was initiated based on these findings.

CT data of the abdominal organs and retroperitoneal space with intravenous contrast showed that the liver parenchyma was totally altered as a result of multiple foci up to 4 cm in diameter with indistinct, uneven contours; contrast enhancement revealed a centripetal pattern of accumulation, which is typical for hemangiomatosis.

Echocardiography revealed a congenital heart defect: a secundum atrial septal defect of 7–8 mm with a left-to-right shunt, mild dilation of the left atrium (z-score +2.34), and pulmonary hypertension (systolic pulmonary artery pressure of 40–43 mmHg). Left ventricular (LV) contractility was satisfactory (LV ejection fraction by Simpson was 72%). The ECG showed sinus rhythm and a normal electrical axis. Analysis of the acid-base status at admission revealed mixed acidosis (pH = 7.119), hyperlactatemia (3.5 mmol/L), and electrolyte disturbances.

Within twelve hours after admission to the ICU, cardiorespiratory monitoring revealed negative dynamics, including depressed consciousness, increasing respiratory failure, and sinus rhythm with a tendency to bradycardia (heart rate dropping to 70 bpm). Treatment included slow intravenous administration of atropine at a dose of 0.01 mg/kg; furosemide—1 mg/kg; and dexamethasone—0.2 mg/kg. Given the progression of respiratory failure, the clinical picture of pulmonary edema, depressed consciousness, and the development of life-threatening bradycardia, the decision was made to initiate mechanical ventilation, which led to stabilization of the child’s condition and normalization of hemodynamic parameters. Analgesic and sedative therapy was initiated with midazolam at 1 µg/kg/min and morphine at 0.02 mg/kg/h. Considering the patient’s profile and the risk of infection with multidrug-resistant pathogens as well as invasive candidiasis, antibacterial prophylaxis was started with a protected beta-lactam antibiotic (cefepime/sulbactam at 80 mg/kg/day based on sulbactam), and antifungal therapy was initiated with fluconazole (12 mg/kg loading dose, followed by 8 mg/kg/day).

The clinical and radiological diagnosis was established based on the clinical presentation and comprehensive examination data: diffuse neonatal hepatic hemangiomatosis with cutaneous involvement; congenital heart defect: secundum atrial septal defect; pulmonary hypertension; grade I circulatory insufficiency; functional class II according to the Ross Classification; and grade 1 respiratory failure. Surgical intervention for the diagnostic morphological verification was contraindicated due to the localization, size, and the severity of the process, as well as the high risks of intra- and postoperative complications including death. Given the benign nature of the lesion, the child was treated with Hemangiol^®^ (propranolol), a non-selective beta-blocker in oral solution form, for total liver involvement by multiple hemangiomas.

Generally, the recommended starting dose is 1 mg/kg/day, divided into two doses of 0.5 mg/kg, with a gradual increase by 1 mg/kg per week up to the therapeutic dose of 3 mg/kg [10]. In our case, due to the patient’s serious condition, the medical consultation recommended the starting drug dose of 0.5 mg/kg/day orally (in three doses), with a gradual daily increase by 0.5 mg/kg/day every 3 days until the maximum therapeutic dose of 3 mg/kg/day was reached. The daily ECG monitoring registered no alterations in the rhythm or conduction.

Propranolol therapy was used along with hormone therapy (L-thyroxine), infusion therapy, prophylactic measures against hemorrhagic complications, hepatoprotective therapy, diuretic therapy, and replacement transfusions of erythrocyte suspension, fresh frozen plasma, cryoprecipitate, 20% albumin solution and single-dose i.v. human immunoglobulin. The replacement transfusions were required during the first 7 days, after which the laboratory parameters returned to normal. From day 3 after normalization of the lactate levels, enteral feeding was performed via a nasogastric tube with an adapted milk formula starting at 5 mL/h with a continuous infusion and gradually increasing to the physiological requirement over 7 days. From day 7, the child received assisted ventilation; the dose of analgesic therapy was gradually reduced.

On day 10 of the therapy, a marked positive trend was registered, evidenced by the resolution of ACS (abdominal circumference: 42 cm; liver palpable 3 cm below the costal margin) and the establishment of effective spontaneous breathing (the child was extubated). On day 14, the child was transferred to a specialized department in stable condition to continue propranolol and concomitant therapy.

Upon transfer to the specialized department, the child was alert, afebrile, and in no apparent distress. Her breathing was spontaneous, effective, and without dyspnea. Blood gas analysis was normal, and oxygen saturation remained stable while breathing ambient air. Hemodynamic status was stable. She was receiving continuous enteral nutrition via nasogastric tube (Nutrilon 1) at a rate of 30 mL/h, with a 4-h rest period at night. Diuresis was adequate while on spironolactone at a dose of 2 mg/kg/day. Bowel movements were spontaneous, with soft, regular stools and no abnormalities. Her weight was 4.3 kg at transfer, compared to 4.5 kg on admission.

The table below presents the patient’s laboratory test results during her stay in the ICU. Treatment resulted in the resolution of severe thrombocytopenia and a significant decrease in ALT levels (Table 1).

A marked positive trend was observed once the maximum therapeutic dose of propranolol (3 mg/kg/day) was achieved. This included a reduction in the size of the facial hemangioma (from 1.5 cm to 0.8 cm in diameter), resolution of ACS (abdominal circumference decreased to 38 cm), normalization of platelet count to within the age-appropriate reference range, and a decrease in TSH, fT3 and fT4 levels. The patient demonstrated satisfactory tolerance of the therapy. No adverse events were recorded throughout the follow-up period.

According to the instrumental examination the dynamics on day 30 of the therapy was as following:

US examination of the liver revealed OTS (subcostal approach, oblique-transverse scanning along the edge of the costal arch at the level of the midclavicular line) reduction to 105 mm, as well as a decrease in the size of multiple focal formations to 8 × 12 mm.

EchoCG showed a reduction in the atrial septal defect to 5–6 mm, normalization of the cardiac chamber dimensions, and no signs of pulmonary hypertension.

MRI of the abdomen demonstrated liver hemangiomatosis with positive dynamics compared to the admission CT. The liver parenchyma contained multiple bulky, irregularly shaped formations (up to 3.7 cm), almost entirely replacing the normal parenchyma. Intravenous multiphase contrast administration revealed centripetal filling of the focal formations (Figure 1a,b).

At discharge from our center, the child continued to receive hormone replacement therapy with L-thyroxine at a dose of 5 µg/kg. A recommendation was given for the follow-up by a pediatric endocrinologist at the place of residence. The thyroid hormone profile during replacement therapy was as follows: TSH remained elevated—18.79 mIU/L (reference range: 0.72–11 mIU/L)—while the levels of thyroxine (fT4)—18.6 pmol/L (reference range: 11.5–28.3 pmol/L)—and triiodothyronine (fT3)—4.7 pmol/L (reference range: 3–4.3 pmol/L)—were within the reference values.

According to the most recent echocardiogram (ECHO-CG) performed at our center, the atrial septal defect persists, with a dynamic decrease in size by 0.1 cm: a left-to-right atrial septal defect measuring 0.4–0.5 cm, which is hemodynamically insignificant. The child was discharged under the supervision of a cardiologist at the place of residence. According to the latest extract from the history of the disease from the place of residence, the child currently has a patent foramen ovale (a 1 mm defect).

At the follow-up control examination after 1 year, the child’s condition was of moderate severity with respect to the main disease. The follow-up MRI of the liver with i.v. contrast revealed significant positive dynamics, manifested by a reduction in the size of multiple bulky lesions with distinct lumpy contours. Both lobes of the liver, previously enlarged due to the lesions, also decreased in size (Figure 2a,b and Figure 3a,b).

Currently, the child’s condition is of moderate severity due to the underlying main disease. The girl continues to receive propranolol therapy at a dose of 3 mg/kg/day at her place of residence. The total duration of follow-up and treatment is 1 year and 9 months as of this writing. The decision to reduce the dose and gradually discontinue the therapy is planned on the basis of the results of a follow-up MRI at our center and is expected in the near future. The hemangioma in the left brow area was surgically removed at the mother’s request for cosmetic reasons. The risk of disease recurrence in the child is extremely low, given the completion of the vascular proliferation phase, which lasts up to a maximum of 8–12 months of life, during propranolol therapy with a gradual reduction in the drug dosage.

## 3. Discussion

Hemangiomatosis of internal organs is rare, but in some cases it carries a severe prognosis, leading to numerous life-threatening conditions; therefore, rapid diagnosis and timely initiation of therapy are critical. Sometimes an individualized approach to dosage titration and adjunctive therapy is required.

Despite the benign nature of hemangiomas, in certain locations and sizes they pose a threat to vital functions, potentially leading to a fatal outcome. The present clinical case is a striking example of the development of a life-threatening condition in diffuse hepatic hemangiomatosis, which was successfully managed with propranolol therapy alongside appropriate supportive care.

Notably, cutaneous IH may be associated with visceral involvement, with the liver being the most commonly affected organ [3,11]. The current classification distinguishes between focal, multifocal, and diffuse hepatic hemangiomas [12].

Diffuse IH of the liver is always accompanied by a severe clinical course that occurs due to ACS, posing a threat to the patient’s life, which requires a multimodal approach to treatment [4,13].

In some cases, hemangiomas may be part of PHACE syndrome (posterior fossa abnormalities; hemangioma; and arterial, cardiac, eye, and sternal abnormalities), including posterior cranial fossa malformation, hemangiomas, heart malformations, and eye malformations. This syndrome is associated with pre-eclampsia or placental abnormalities [13,14]. Another syndrome manifested by hemangiomas is lumbar spine syndrome—LUMBAR (lumbar spine syndrome—multiple hemangiomas of the lower body, developmental abnormalities of the genitourinary system, bone deformities, and vascular malformations) [13,14,15,16]. A number of patients with diffuse hemangiomatosis with affected liver, had persistent thrombocytopenia and hypocoagulation, which was associated with Kazabach—Merritt syndrome [13,14,15,16]. Consumptive hypothyroidism may also develop, requiring hormone replacement therapy, but this complication resolves as the tumor involutes; therefore, the drug dosage is reduced until complete withdrawal [17].

The diffuse hemangiomatous lesions are often accompanied by hypothyroidism, probably due to the high levels of type 3 iodothyronine deiodinase activity produced by hemangiomas [18]. According to Kassarjian et al. [19], hypothyroidism in patients with infantile hepatic hemangiomas (IHH) is a poor prognostic factor correlating with an increased risk of complications including sepsis, multiple organ failure, pulmonary hypertension, and intracranial hemorrhage. These patients are more likely to have a massive tumor burden, leading to compression of neighboring organs and vessels, rather than the more common pattern of high-output congestive heart failure. Obviously, in patients with extensive infantile hepatic hemangioma, thyroid function should be evaluated.

In the present study, the patient with an advanced IHH with no morphological changes in the thyroid gland had signs of hypothyroidism that manifested in a decrease in the level of the thyroid hormones. The patient received thyroxine replacement therapy on the basis of these data.

In early studies, corticosteroids in combination with digoxin and diuretics were used as the first line for IHH treatment [20]. However, side effects such as stomach irritation, osteoporosis, psychological disorders, and abnormal sedimentation limited the duration of this strategy. In addition, a significant proportion of patients (23.1%) was resistant to steroids [21]. In the past, interferon α-2 (INFα-2) was used in cases that could not be treated with steroids, as it inhibited the proliferation and migration of endothelial cells, causing a decrease in the size of intracranial hypervascular formation [22]. However, INFα could cause serious complications such as spastic diplegia and developmental disorders in children under 3 years of age. INFα is no longer recommended in current guidelines for the treatment of hypervascular formations [23].

In 2008 Leaute-Labreze et al. [24] reported the results of a study where they accidentally discovered that propranolol was a non-selective antagonist of β-1 and β-2 adrenergic receptors, and showed anti-proliferative effect in infantile hemangioma. Later, French researchers designed a drug for IH treatment in infants on the base of propranolol in the form of an oral solution, Hemangiol^®^. Currently, the therapy with this drug is recommended as the therapy of choice for patients with IH who require systemic therapy [24,25,26]. Hemangiol^®^ (international non-patented name (INN)—propranolol) is the only officially registered drug for IH treatment in children in Europe and the USA, which is included in European, American and Russian clinical guidelines for the treatment of this disease [26,27]. The drug was registered in Russia in 2015 and is also successfully used in clinical practice in children from 5 weeks of life [1,2].

The exact mechanisms underlying propranolol’s potential to cause hemangioma involution are unclear. This phenomenon may be associated with vasoconstriction mediated by pericytes, a decrease in the fibroblast growth factor levels, and inhibition of the renin-angiotensin system [28].

The FDA approved propranolol for the systemic therapy of idiopathic hyperhidrosis in 2014. After the approval, propranolol has been used safely and effectively for idiopathic hyperhidrosis and has substituted steroid agents [29]. The recommended propranolol therapy regimen was established from the starting dose of 0.6 mg/kg two times a day and a progressive increase to the target of 1.7 mg/kg two times a day. Another regimen, which was suggested by the European Expert Group, was 1.0 mg/kg/day reaching 2.0–3.0 mg/kg/day [23]. However, the dosage should be adjusted to the patient’s condition, especially where physiological abnormalities or sleeping disorders are observed as mentioned by Kulungowski et al. [8,30]. It is important that treatment finishes half a year before the child reaches the age of 12 months. During the period of dose reduction or after discontinuation of the drug therapy, 10–25% of patients may experience rebound growth of IH. A study showed that the patients who discontinued the therapy at the age of less than one year had the highest risk of recurrence, while the lowest risk was observed in patients whose treatment was discontinued between 12 and 15 months of age [13].

Despite the fact that hemangioma is a benign neoplasm, the diffuse form of the disease with massive hepatic involvement is associated with mortality rate of approximately 70% [3,11,25]. Rapidly growing lesions can lead to life-threatening conditions as described by Rialon et al. [31]. Multiple lesions can also lead to platelet accumulation in tumors, which causes anemia, thrombocytopenia, and coagulopathy [32]. High mortality is associated with the development of multiple organ failure in patients with ACS, as well as with DIC (disseminated intravascular coagulation syndrome) in case of coagulopathy with liver damage. In some cases, although US analysis and MRI usually allow for an accurate differential diagnosis, it may be difficult to diagnose a pathological lesion. Differential diagnosis of a hepatic hemangioma should be distinguished from metastatic or atypical neuroblastoma, hepatoblastoma, mesenchymal hamartoma, kaposiform hemangioendothelioma, embryonal sarcoma, or angiosarcoma. Thus, taking into account the potential for developing a malignancy, thorough diagnostic examination and disease monitoring are ultimately important. IH can be diagnosed based on clinical characteristics and US/MRI analysis [33]. US examination is preferable, while MRI is recommended in case of an unclear diagnosis. CT scanning is not recommended due to its lower resolution, which presents a higher risk of irradiation in children. In rare cases, a diagnostic biopsy of the lesion may be required. Biopsy may be performed if clinical and imaging findings are atypical [34]. Treatment is primarily aimed at stimulating tumor involution to reduce the lesion size, resolve LTC, and prevent compression of vital organs.

In our case, clinical data and MRI results allowed a diagnosis of IH and did not require a fine-needle aspiration biopsy to prove the diagnosis. Thus, monitoring tumor markers and careful study of the disease history and imaging data, in particular the structural features of the tumor and the degree of contrast accumulation, made it possible to exclude other malignancies. A biopsy of the tumor can be crucial in establishing a diagnosis, but it is not always possible, including this case, given our patient’s extremely serious condition. The clinical response to propranolol therapy fully confirmed the diagnosis in our case, which could not be possible in other diseases.

The clinical presentation in this case was generally consistent with the previously described clinical cases. In particular, Li at al. reported a similar case of an efficient therapy of a 2-month old infant with IH by propranolol in a maximal therapeutic dose of 2 mg/kg/day [34]. However, in our case, considering the infant’s life-threatening condition, the maximal dose was enhanced up to 3 mg/kg/day with no significant adverse reactions.

The remaining unresolved issue is the choice of the appropriate therapy. Symptomatic treatment of cardiac failure, hypothyroidism, and hemocytopenia can be used in cases of diffuse hemangiomatosis with liver involvement that do not cause life-threatening conditions [32].

The development of life-threatening conditions in diffuse hemangiomatosis can be determined not only by the volume or size of the lesions but also by their location. In these cases, propranolol, which has demonstrated efficacy in the treatment of IH, is the drug of choice. The presented clinical case demonstrated the effectiveness of long-term propranolol use in the treatment of life-threatening conditions in the patient with IH.

## 4. Conclusions

Visceral hemangiomas in newborns are rare and potentially life-threatening conditions that require a multidisciplinary approach to diagnosis and treatment. Concomitant conditions must be carefully considered, and treatment should be monitored in collaboration with a cardiologist and other relevant specialists. In the early stages of treatment, it may also be necessary to consult an intensivist to assess and manage patients with LTC with organ system dysfunction. Timely detection and adequate treatment of infantile hemangiomas significantly improve the prognosis and quality of life for these patients.

## Figures and Tables

**Figure 1:**
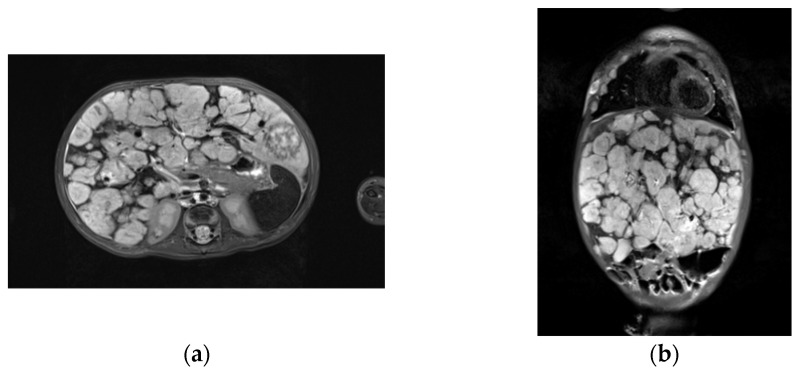
MRI images of the patient’s liver on day 30 of the therapy. T2-weighted images in axial (**a**) and coronal (**b**) projections present an increase in the size of both lobes of the liver due to multiple volumetric formations with clear bumpy contours. The structure of the formations is moderately heterogeneous and solid.

**Figure 2:**
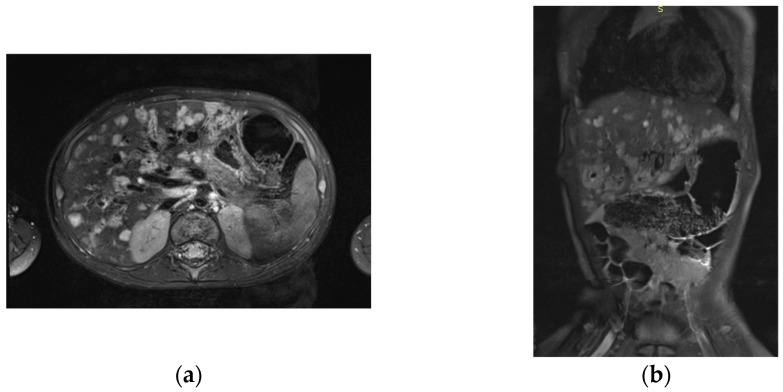
MRI images of the patient’s liver one year after propranolol therapy. T2-weighted axial (**a**) and coronal (**b**) MR images demonstrate significant improvement following propranolol therapy. There is a reduction in the size of the multiple solid mass lesions, which have distinct, nodular contours. Both liver lobes, previously enlarged, have also decreased in size. The internal structure of the masses remains unchanged, appearing moderately heterogeneous and solid.

**Figure 3:**
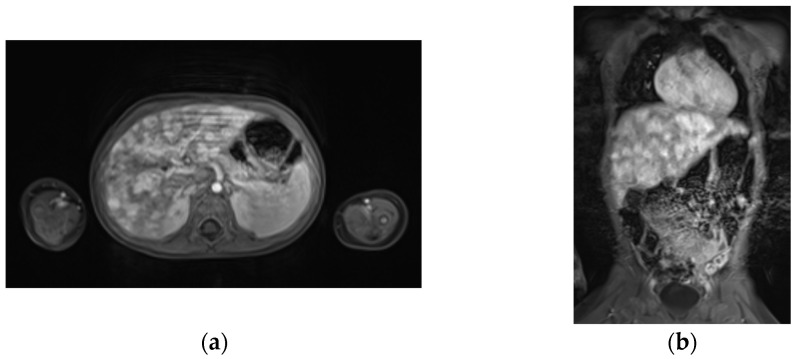
MRI images of the patient’s liver one year after propranolol therapy. T1-weighted axial (**a**) and coronal (**b**) images obtained during multiphase contrast enhancement still demonstrate intense centripetal filling of the largest lesions by the contrast agent; smaller nodules accumulate contrast agent throughout their entire volume during the arterial phase of contrast enhancement. The liver lesions continue to show no diffusion restriction on DWI (b = 800 s/mm^2^).

**Table 1:** The dynamics of the laboratory tests during treatment over the first 14 days in the ICU.

Day/ Parameter	1	2	3	4	5	6	7	8	9	10	11	12	13	14	Ref. Range
Hemoglobin, g/L	89	86	131	150	141	131	124	127	108	90	87	87	87	87	115–175
Platelets, 10^9^/L	48	60	82	50	43	86	100	151	182	237	231	256	272	310	210–560
INR	1.42	1.69	1.76	1.49	1.51	1.32	1.24	1.31	1.29	1.21	1.3	1.26	1.36	1.33	0.85–1.1
Fibrinogen, g/L	0.4	1.48	1.7	1.2	1.3	2.2	2.7	3.2	3.2	3.0	2.4	2.2	2.3	2.0	1.8–3.5
Total bilirubin, µmol/L	89.8	80.7	147.7	215.8	186.3	157.4	130.2	117.3	97.0	76.6	56.6	53.4	46.8	36.0	1.7–20.5
ALT, mol/L	164.1	165.1	149.7	94.3	55.8	59.5	46.8	57.4	63.9	59.2	56.3	68.8	61.6	53.0	<45

Note: INR—international normalized ration; ALT—alanine aminotransferase.

## Data Availability

The original contributions presented in this study are included in the article. Further inquiries can be directed to the corresponding author.

## Abbreviations

The following abbreviations are used in this manuscript:

ACS	abdominal compartment syndrome
AFP	α-fetoprotein
ALT	alanine aminotransferase
CT	computed tomography
DIC	disseminated intravascular coagulation syndrome
ECG	electrocardiography
FDA	Food and Drug Administration
i.v.	intravenous
ICU	intensive care unit
IH	infantile hemangioma
IHH	infantile hepatic hemangiomas
INFα-2	interferon α-2
INN	international non-patented name
LTC	life-threatening conditions
LV	left ventricular
MRI	magnetic resonance imaging
NSE	neuron-specific enolase
OTS	oblique-transverse scanning
TSH	thyroid-stimulating hormone
US	ultrasound
β-HCG	β-chorionic gonadotropin
